# Animal venoms: a novel source of anti-*Toxoplasma gondii* drug candidates

**DOI:** 10.3389/fphar.2023.1178070

**Published:** 2023-05-03

**Authors:** Dongqian Yang, Xiaohua Liu, Jing Li, Jing Xie, Liping Jiang

**Affiliations:** ^1^ Department of Parasitology, Xiangya School of Medicine, Central South University, Changsha, Hunan, China; ^2^ China-Africa Research Center of Infectious Diseases, Xiangya School of Medicine, Central South University, Changsha, Hunan, China

**Keywords:** *Toxoplasma gondii*, animal venoms, venom peptides, potential mechanism, perspective

## Abstract

*Toxoplasma gondii (T. gondii)* is a nucleated intracellular parasitic protozoan with a broad host selectivity. It causes toxoplasmosis in immunocompromised or immunodeficient patients. The currently available treatments for toxoplasmosis have significant side effects as well as certain limitations, and the development of vaccines remains to be explored. Animal venoms are considered to be an important source of novel antimicrobial agents. Some peptides from animal venoms have amphipathic alpha-helix structures. They inhibit the growth of pathogens by targeting membranes to produce lethal pores and cause membrane rupture. Venom molecules generally possess immunomodulatory properties and play key roles in the suppression of pathogenic organisms. Here, we summarized literatures of the last 15 years on the interaction of animal venom peptides with *T. gondii* and attempt to explore the mechanisms of their interaction with parasites that involve membrane and organelle damage, immune response regulation and ion homeostasis. Finally, we analyzed some limitations of venom peptides for drug therapy and some insights into their development in future studies. It is hoped that more research will be stimulated to turn attention to the medical value of animal venoms in toxoplasmosis.

## 1 Introduction

*Toxoplasma gondii* (*T. gondii*), an obligate intracellular parasite, can widely infect warm-blooded animals. And is considered one of the most successful parasites. Although infection with *T. gondii* in healthy people is mostly asymptomatic, infection in patients with immunodeficiency shows severe symptoms and a poor prognosis. *Toxoplasma* encephalitis in patients with acquired immunodeficiency syndrome (AIDS) is life-threatening due to the reactivation of tissue cysts in the brain (Abgrall et al., 2001). The neurotropic parasite also deepens the mental illness in schizophrenia and bipolar disorder by affecting systemic changes in the brain and peripheral tissues (Kano et al., 2020). Infection in pregnant women causes congenital toxoplasmosis, which usually leads to abortion, fetal malformations, and visual and intellectual developmental disorders (Lopez et al., 2000).

The life cycle of *T. gondii* consists of two stages: sexual reproduction and asexual reproduction. The feline is the definitive host of *T. gondii*. And their intestine provides a suitable environment for sexual phases. Ingested bradyzoites develop into merozoites, which can differentiate into macrogametes and microgametes. They subsequently fuse to form oocysts and are eventually excreted in feces. Oocysts become infectious forms containing sporozoites in the environment (Martorelli Di Genova and Knoll, 2020). *T. gondii* has two infective forms in the intermediate host. The tachyzoite with a complex lytic cycle is highly invasive. It initiates the invasion process with the help of conoid and secretory organelles (micronemes and rhoptries) (Paredes-Santos et al., 2012). Successful invasion needs to form an adhesive bridge and form moving junction (MJ). Then the host cell membrane invaginates and provides a parasitophorous vacuole (PV) for parasite survival. Another secretory organelle, dense granule, releasing proteins decorates the parasitophorous vacuole membrane (PVM) to evade host cell assaults (Sibley, 2004; Mercier and Cesbron-Delauw, 2015). Within its PV, *T. gondii* keeps asexual replication until egress. Once tachyzoites successfully evade host immunity attack, they transform into bradyzoites and settle into long-lived cells such as neurons as tissue cysts (Cabral et al., 2016).

**FIGURE 1:**
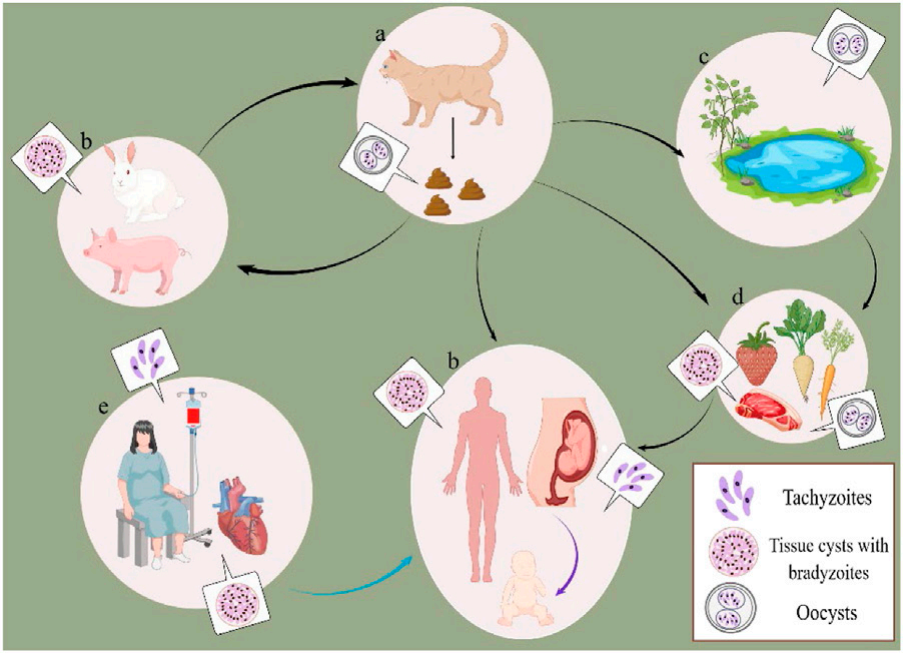
Common infection routes of *T. gondii*. **(A)** the definitive host. **(B)** the intermediate host. **(C)** oocyst-contaminated environment. **(D)** food contaminated with oocysts or tissue cysts. **(E)** blood or organ containing tachyzoites or tissue cysts. The black line: horizontal transmission. The purple line: transplacental transmission. The blue line: blood and organ transplant transmission.

*T. gondii* can be transmitted through a variety of routes (Figure 1). Firstly, the intermediate host is infected by ingesting tissue cysts (containing bradyzoite) in undercooked meat, eating or drinking oocyst-contaminated food/water, or obtaining oocyst from cleaning the cat litter either via ingestion or inhalation. The congenital infection is caused by transplacental transmission. Furthermore, humans who receive blood transfusion and organ transplantation, which may contain tachyzoites or tissue cysts, are vulnerable to infection (Paduan et al., 2018; Coelho et al., 2020; Garweg et al., 2022).

One of the current therapies for toxoplasmosis is the combination of sulfadiazine and pyrimethamine (SDZ-PYR), which can effectively resist *T. gondii*. But these drugs can cause side effects including hematological reactions, hypersensitivity, gastrointestinal disorders, drug resistance, and even damage to the liver and kidney (Doliwa et al., 2013). Pyrimethamine is an antagonist of folic acid. It blocks the dihydrofolate reductase (DHFR) of *T. gondii* and also affects DNA synthesis and cell proliferation. Clindamycin is often considered as an alternative treatment for patients with SDZ-PYR allergy as well as intolerance. However, it is less effective for long-term treatment and for preventing relapse (Katlama et al., 1996). For the treatment in early pregnancy within 16 weeks, spiramycin is the preferred choice which inhibits parasite protein synthesis. But it has poor permeability in brain tissue while not being used to treat fetal infection due to its toxic effects (Hotop et al., 2012). Trimethoprim and sulfamethoxazole can cross the placental barrier but increase the risk of fetal teratogenicity (Masters et al., 2003). In addition, sulfonamide resistant strains have been reported (Meneceur et al., 2008). Atovaquone and azithromycin can be used for the treatment of tissue cysts, but drug resistance and low penetration lead to poor efficacy (Montazeri et al., 2018). Overall, current drugs for toxoplasmosis still present challenges. Therefore, there is still a need to find more valuable drugs.

## 2 Animal venoms

The question of “curse or cure?” is a fascinating one for venomous animals. Statistics show that there are some 220,000 species of toxic creatures around the world, with a wide distribution from the sea to the land and deserts (Holford et al., 2018). Previous studies have been trying to investigate how these venoms can be used in medical research since ancient times. This idea can even be traced back thousands of years. Blood-sucking leeches are widely used for the treatment of systemic diseases (Whitaker et al., 2004). Recently, the active peptide Ppnp7 in the venom of social wasp *Polybia paulista* has also been shown to have possible therapeutic value in epilepsy (Silva et al., 2020). To date, a variety of animal venom-derived substances have been used in clinical disease treatment. Exenatide is a GLP-1 receptor agonist extracted from the saliva of the *Gila monster lizard* and is used in the treatment of type 2 diabetes (Pradhan et al., 2020). Ziconitide, derived from *Cone snail*, targets the CaV2.2 channel for the treatment of chronic pain (Miljanich, 2004). In addition, Captopril, a Pit viper extract for the treatment of hypertension has received clinical approval (Cushman and Ondetti, 1991). Antivenom can be used to neutralize the neurotoxins, circulating toxins and cytotoxins in the venoms for human treatment. Some poisonous substances with neuroactive properties can block the interaction between neurotransmitters and receptors. For example, blocking glutamate receptors is a major application of animal venoms in analgesia (Monge-Fuentes et al., 2018).

Subsequent researches have revealed that animal venoms contain a variety of bioactive components based on proteomic analysis. The components are complex but similar from species to species which mainly consist of peptides as well as some proteins (Ghezellou et al., 2022; Wu et al., 2023). Cationic peptides rich in α helix or β-Sheet structure are promising candidates for combating various bacteria and parasites (Borges et al., 2006; Li et al., 2014). In addition, previous studies have shown that some peptides can target a variety of membrane ion channels (Miljanich, 2004). Other defensins can affect protein synthesis by inhibiting DNA replication. It also enhances the host immune response to inhibit the virus and other pathogenic organisms (Chen et al., 2012). Regarding other substances, analysis of rattlesnake venom has revealed that crude venom contains up to 20 protein families (Deshwal et al., 2021). These include the metalloproteases and phospholipases (PLA2), cysteine-rich secretory proteins (CRISPs), C-type lectin and so on. These have shown various functions in regulating ion channels, mediating inflammatory responses (Castanheira et al., 2015). Snake venom PLA2 (svPLA2) affected membrane instability and permeabilization by targeting bacterial membrane, and protein synthesis by targeting bacterial ribosomes (Samy et al., 2017). Along with the substances pointed above, venoms are rich in a variety of lipid components with the functions in metabolism and energy homeostasis, such as cardiolipin (CL) and triacylglycerol (TG). Regretfully, we did not detect relevant research about their functions in anti-*T. gondii* (Ghezellou et al., 2022).

## 3 Animal venoms and venom-derived molecules with anti-*T. gondii* activity

Here, we used PubMed as the medical database source and focused on the medicinal value of animal-derived molecules, crude venoms, against *T. gondii.* (Table 1).

**TABLE 1:** Animal venoms and venom-derived molecules currently used in the anti-*T. gondii* research.

Candidate name	Animal’s name	Category	Effects	Safe dose range with activity	References
Longicin P4	*Haemaphysalis longicornis* (hard tick)	Peptide	Inducing tachyzoites aggregation and membrane disruption; inhibiting invasion and replication	5–50 μΜ	Tanaka et al. (2012)
S-cal14.1a	*Conus californicus* (snail)	Peptide	Inhibiting tachyzoites invasion and replication	10–50 μΜ	De León-Nava et al. (2016)
BnSP-7	*Bothrops pauloensis* (snake)	Protein	Inhibiting tachyzoites adhesion and replication; modulating host immune response	12.5–50 μg/mL	Borges et al. (2016)
BpLec	*Bothrops pauloensis* (snake)	Protein	Inhibiting tachyzoites adhesion and replication; modulating host immune response	2.5–10 μg/mL	Castanheira et al. (2015)
Lycosin-I	*Lycosa singorensis* (spider)	Peptide	Inhibiting tachyzoites invasion and replication; extending mice survival rate	TC_50_ = 34.69 μΜ IC_50_ = 10.08 μΜ	Tang et al. (2019)
XYP1	*Lycosa coelestis* (spider)	Peptide	Inhibiting tachyzoites invasion and replication; extending mice survival rate	IC_50_ = 38.79 μΜ	Liu et al. (2021)
*Tityus serrulatus*	Scorpion	Crude venom	Inhibiting tachyzoites replication; modulating host immune response	25–100 μg/mL	de Assis et al. (2021)
HWVM	*Ornitoctonus huwena* (spider)	Crude venom	Reducing tachyzoites invasion and intracellular replication rates; extending mice survival rate	3.125–12.5 μg/mL	Hou et al. (2019)
JZVM	*Chilobrachys jingzhao* (spider)	Crude venom	Reducing tachyzoites invasion and replication rates	6.25–12.5 μg/mL	Hou et al. (2019)
*Hemiscorpius lepturus* venom	*Hemiscorpius lepturus*	Crude venom	Inhibiting tachyzoites viability	IC_50_ = 39.06 μg/mL CC_50_ = 72.46 μg/mL SI = 1.855	Khaleghi Rostamkolaie et al. (2019)
Neuwiedase	*Bothrops neuwiedi* (snake)	Protein	Reducing tachyzoites invasion and replication rates	0.7–12 μg/mL	Bastos et al. (2008)

TC_50_, the median toxic concentration; IC_50_, the half maximal inhibitory concentration; CC_50_, half cytotoxic concentration; SI, selectivity index (CC_50_/IC_50_).

The longicin P4 peptide (SIGRRGGYCAGIIKQTCTCYR) is a partially defensin-like peptide isolated from *Haemaphysalis longicornis* (Tanaka et al., 2012). The molecular weight (MW) and isoelectric point (pI) are 2306.7Da and 9.50 separately. Other peptides, P1, P2 and P3, were also isolated, but only the P4 peptide showed antibacterial and anti-*Babesia sp*. activity (Tsuji et al., 2007). The P4 peptide is sourced from the C-terminal region of longicin containing arginine and lysine. Tanaka et al. evaluated its inhibitory effects on *T. gondii* (Tanaka et al., 2012). Trypan blue stain showed that it induced the parasite accumulation after 5 min treatment and the mortality rate is as high as 90% after 1 h. Scanning electron microscope (SEM) showed that it effectively disrupted *T. gondii* membrane homeostasis, causing morphological changes such as membrane ultrastructure disruption and cavitation as well as swelling. As observed by transmission electron microscope (TEM), treatment with 50 µM P4 peptide induced organelle disruption and triggered leakage of cellular contents. They speculated that this class of peptides may inhibit parasite survival by targeting the cell membrane.

S-cal14.1a (GDCPPWCVGARCRAEKC) is a cysteine-rich synthetic peptide isolated from *Conus californicus* with significant activity against bacteria and cancer (Biggs et al., 2010). De León-Nava MA and co-workers evaluated its effects on *T. gondii* invasion, proliferation, viability and virulence *in vitro* (De León-Nava et al., 2016). At micromolar concentrations, s-cal14.1a reduced tachyzoites viability in a dose-dependent manner. At 10 nM concentration, viability and invasion rates of *T. gondii* were lowered by approximately 50% and 61%, respectively, and the inhibition effect was more pronounced with increasing toxin concentration. Therefore, they hypothesized that S-cal14.1a was able to penetrate the PVM and the parasite plasm membrane to disrupt the replication. S-cal14.1a is a structured peptide with cell membrane as well as ion channel targeting (De León-Nava et al., 2016). Large conotoxins can distinguish between pathogenic and human ion channel subtypes and have their own corresponding ligand binding sites. The selective target of ion channels to destroy *T. gondii* explains its low toxicity to host cells.

BnSP-7 toxin is a homologous protein of Lys49 PLA2 isolated from the venoms of *Bothrops pauloensis* (Soares et al., 2000). PLA2 has been studied for its medicinal value in viral inhibition and anti-parasitism (Siniavin et al., 2021). It has been reported that PLA2 can damage the parasite plasm membranes and cause changes in membrane composition and extravasation of proteins. BnSP-7 had anti-*Leishmania* activity with half maximal inhibitory concentrations (IC_50_) value of 58.7 μg/mL. BnSP-7 (200 μg/mL) interfered with parasite proliferation and induced changes in membrane protein profile (Nunes et al., 2013). In *T. gondii*, BnSP-7 at different concentrations (12.5–50 μg/mL) did not inhibit parasite mortality but reduced its invasion and proliferation (Borges et al., 2016). Researchers speculated that BnSP-7 may target the parasite plasma membrane and reduce its invasion by disturbing membrane homeostasis. Proliferation is reduced by affecting proteins on the plasma membrane possibly. This inference is consistent with the mechanism of PLA2. ELISA experiment showed that BnSP-7 induced upregulation of Interleukin-6 (IL-6) and macrophage migration inhibitory factor (MIF), representing that BnSP-7 can stimulate the expression of pro-inflammatory factors. Modulating host immune response is an important mechanism against *T. gondii*.

BpLec is a C-type lectin isolated from *Bothrops pauloensis* snake venom (Castanheira et al., 2013). It exerts bioactive effects by combing with a variety of substances, such as galactose, to form a calcium ligand ring, and cell membranes in a carbohydrate-specific manner. Its functions in antibacterial, antileishmanial, angiogenic and inflammatory regulation have been demonstrated (Castanheira et al., 2013; Castanheira et al., 2017). Castanheira et al. (2015) evaluated its effects against *T. gondii*. It was able to reduce the invasion at safe concentrations (2.5–10 μg/mL), but its inhibitory effect could be rescued by galactose. They hypothesized that this might be due to the reduced adhesion through the interaction of BpLec with glycoproteins on the parasite membrane. In terms of immunomodulation, BpLec affected the immune response of host cells by inducing upregulation of pro-inflammatory factor (IL-6 and MIF) and balancing levels of anti-inflammatory factor transforming growth factor-β1 (TGF-β1) in HeLa cells.

Lycosin-I (RKGWFKAMKSIAKFIAKEKLKEHL, Mw:2732.37Da, pI: 10.13) and XYP1 (KIKWFKAMKSIAKFIAKDQLKKHL, Mw: 2900.643Da, pI: 10.48), with 66.6% amino acid sequence similarity and typical α-helix structure, are amphiphilic cationic antimicrobial peptides isolated from spider venoms (Tang et al., 2019; Liu et al., 2021). Both natural or modified Lycosin-I exhibited an extensive range of bioactivities such as anti-inflammatory, anti-tumor and antibacterial (Zhang et al., 2017; Li et al., 2018). Amino acid modification or substitution of Lycosin-I could significantly improve its serum stability and antitumor activity (Jian et al., 2018). Tang et al. (2019) and Liu et al. (2021) have confirmed that Lycosin-I and XYP1 shown anti-*T. gondii* activity at concentration of 10 µM. Compared to Lycosin-I, XYP1 was shown to have better anti-*T. gondii* activity and lower cytotoxicity to human foreskin fibroblast cells (HFFs). Both of them appeared to have more significant effects on invasion and proliferation compared to SDZ. They speculated that this mechanism may be related to the interaction of the peptide with membrane proteins of *T. gondii*. In addition, XYP1 (4 mg/kg) extended the survival time of infected mice. Lycosin-I pretreatment prolonged the survival time by approximately 50 h. SEM experiment showed that both spider peptides appeared to directly trigger the parasite membrane, causing membrane morphological changes including loss of the crescent shape, swelling of tachyzoites, membrane wrinkling and rupture.

*Tityus serrulatus* venom (TsV) is a mixture of active components from scorpion with immunomodulatory properties (de Assis et al., 2021). It has been extensively studied for its pro-inflammatory, T-lymphocyte activation which can stimulate the expression of inflammatory factors, such as interleukins, tumor necrosis factor (TNF) and interferon-γ (IFN-γ) (Zoccal et al., 2011; Casella-Martins et al., 2015). De Assis et al. (2021) extracted scorpion venom and distilled using chromatography to obtain seven subgroups (F1, F2, F3, F4, F5, F6, and F7). About the anti-*T. gondii* activity *in vitro* of these seven subgroups, only F6 and F7 induced nitric oxide (NO) production in macrophages (MOs) at safe concentrations and exerted biological activity. The F6 fraction was further fractionated into Sub6-A and Sub6-B and sequenced for comparison. Sub6-B showed 93% sequence similarity to the toxin Ts2. Ts2 is capable of inflammatory regulation *in vivo*, which implied that Sub6-B, the major part of F6, had significant anti-*T. gondii* as well as immunomodulatory capacity. They suggested that TsV does not target the membrane, but rather promotes the activation of MOs and the release of inflammatory mediators such as IL-12 and TNF.

The spider venoms HWVM and JZVM, extracted from the venoms of two spiders respectively, *Ornitoctonus huwena* (*O. huwena*) and *Chilobrachys jingzhao* (*C. jingzhao*), displayed anti-*T. gondii* activity (Hou et al., 2019). The spider venoms isolated from them have been shown to exhibit anti-inflammatory as well as sodium ion (Na^+^) channel activating activity (Xiao et al., 2007; Ning et al., 2013). Hou et al. (2019) investigated the activity of these two venoms against *T. gondii in vivo* and *in vitro*. On the one hand, HWVM and JZVM provided moderate anti-*T. gondii* activity in a time-dependent manner. After exposure to HWVM and JZVM for 12 h, survival rates of tachyzoites were 21.2% and 32%, respectively. However, in the acute infection model, JZVM was not as effective as HWVM in prolonging the survival time of infected mice. All mice in the JZVM-treated or SDZ-treated groups died at 12 days, while 50% in the HWVM-treated group survived until 17 days. On the other hand, both showed low cytotoxicity at 12.5 μg/mL. However, with increasing venom concentration, the compounds were significantly more toxic to HeLa cells. Although both venoms have shown anti-parasitic effects *in vivo* and *in vitro*, the active substances in the venoms and the mechanism need to be further investigated.

The anti-*T. gondii* activity of Hemiscorpius lepturus venom *in vitro* was described in a study by Khaleghi Rostamkolaie et al. (2019). It has been shown that the venom contains 12% of the peptides with antibacterial activity. Interaction between venom with parasite showed a significant reduction in *T. gondii* viability in a concentration-dependent manner. It showed lower cytotoxicity and higher anti-*T. gondii* activity compared to SDZ. The CC50 value and IC50 values of the venom were 72.46 and 39.06 μg/mL *in vitro*, respectively, while its mean lethal doses (LD50) value was 6.3 mg/kg in mice.

Neuwiedase, a metalloprotease, was first extracted from the *Bothrops neuwiedi* venom (Rodrigues et al., 2000). Bastos et al. (2008) evaluated its effects against *T. gondii in vitro*. They carried out a comprehensive assessment of neuwiedase by cytotoxicity assay, measurement of cytokine production and analysis of *T. gondii* invasion and replication. The results showed that neuwiedase (12 μg/mL) was effective in inhibiting invasion with inhibition rate by up to 71%. At the same concentration, it did not have a significant effect on the intracellular proliferation. When the parasite pre-treated with neuwiedase infected HFFs, the replication rate was inhibited. TC_50_ value was 12.48 μg/mL. They speculated on two possibilities. One was that neuwiedase was likely to act at the time of cell lysis. By degrading laminin, a bridge for *T. gondii* to invade host cells, the invasion rate was reduced. Alternatively, it was able to hydrolyse certain proteins on the parasite plasma membrane after co-incubation. These proteins were able to affect the formation of the PVM thereby inhibiting proliferation. However, these assumption mechanisms still require further validation.

## 4 Potential biological mechanism of venom peptides against *T. gondii*

### 4.1 Destruction of *T. gondii* cell membrane

The invasion process relies on the formation of MJ following membrane contact. *T. gondii* relies on the PVM to exchange substances with the external environment, absorb nutrients and secrete proteins to maintain its reproduction (Guerin et al., 2017). Mass spectrometry revealed that the *T. gondii* membrane is composed of multiple functional proteins that manipulate the parasite motility, such as endosomal complexes, surface antigens (SAGs), ROPs, micronemes (MICs), cytoskeleton-associated proteins and several proteins associated with metabolism (Cruz-Miron et al., 2021). Some of them are promising drug targets such as ROPs associated with invasion as well as intracellular proliferation (El Hajj et al., 2007).

Through SEM, it found that venoms could induce membrane damage, which suggested venoms may target membrane against *T. gondii*. Firstly, some protein expression is closely related to the membrane biogenesis of *T. gondii*. The inner membrane complex (IMC) of *T. gondii* consists of flattened alveoli arranged around the parasite body, subpellicular microtubules and some intramembranous particles. It is known that its construction are clathrin-coated vesicles which produced by the ER-Golgi secretory pathway (Pieperhoff et al., 2013). A remarkable function of the IMC is in maintaining the crescent shape of tachyzoite and in overcoming mechanical resistance during invasion (Ouologuem and Roos, 2014). Palmitoyl acyltransferases with conserved Asp-His-His-Cys (DHHC) motifs are associated with the function of IMCs. Study has shown that TgDHHC deficiency caused the disintegration of the IMCs as well as morphological changes (Dogga and Frenal, 2020). In addition, an adequate source of phospholipids is essential for PVM and intracellular proliferation. *T. gondii* membrane contains a variety of phospholipid components. These phospholipids are usually widely and asymmetrically distributed in the bilayer and essential for tachyzoites invasion as well as the formation of progeny. Phospholipid production in *T. gondii* relies on two main pathways: self-synthesis and the robbing of lipid components from the host cell (Gupta et al., 2005; Ren et al., 2020). The latter way is dependent on phospholipid-flipping enzymes in the parasite membrane. Lipids from the environment enter into the parasites by binding to the flippase such as ATP-dependent translocases (Chen et al., 2021). A total of five P4-ATPases (1–5) were detected in *T. gondii*. TgP4-ATPase2/5 is located on the parasite membrane and TgP4-ATPase1-3 damaged by auxin induced parasite proliferation inhibition (Chen et al., 2021). Currently, a precise description of how venoms influence membrane biogenesis has not been studied and therefore needs to be demonstrated in future studies.

In addition, this membrane damage, as well as the abnormal membrane shape, may be related to the structure of the venom-derived molecules. Some venom peptides are amphiphilic cationic peptides with amphiphilic α helix structure, which is essential for targeting the plasma membrane of *T. gondii*. The direct interaction with the parasite membrane leads to changes in the morphology of the membrane and even to severe porosity as well as membrane rupture and leakage of contents (Samy et al., 2017). A detailed mechanisms description of peptide-mediated membrane disruption is provided by Baxter et al. (2017). They summarized three action models including the carpet model, the toroidal pore model and the barrel-stave model. The latter two models involved membrane pore formation as well as insertion of α-helical peptides. Different concentrations of XYP1 and Lycosin-I induced membrane invagination, membrane pores and tachyzoites swelling. Longicin P4 peptide exhibited a high affinity for biological membranes which can induce membrane damage through electrostatic interaction. These phenomena reinforce our hypothesis that venom peptides accumulated on *T. gondii* plasm membrane and formed pores through single or multiple models. Although no mechanistic studies have been conducted on BnSP-7, a comprehensive mechanism has been elucidated for its homologue, PLA2. This class of proteins has two independent action sites: a cationic membrane-docking site (MDoS) at the C-terminus and a hydrophobic membrane disruption site (MDiS) consisting of highly hydrophobic residues (Leu and Phe) (Fernandes et al., 2013). The electrostatic action of the protein in contact with the membrane induces the exposure of MDoS and MDiS and the rearrangement of the phospholipid structure in the membrane. MDiS inserts directly into the lipid bilayer and reduces membrane permeability ultimately disrupting membrane integrity (Bortoleto-Bugs et al., 2007; Montecucco et al., 2008). In addition, *T. gondii* possesses a variety of glycoproteins that regulate the adhesion and invasion process (Luo et al., 2011). BpLec possesses a carbohydrate recognition domain (CRD) in the C-terminus. It can interfere with the adhesion by targeting some glycoconjugates on the parasite membrane (Castanheira et al., 2015).

### 4.2 Regulation of host immune response

**FIGURE 2:**
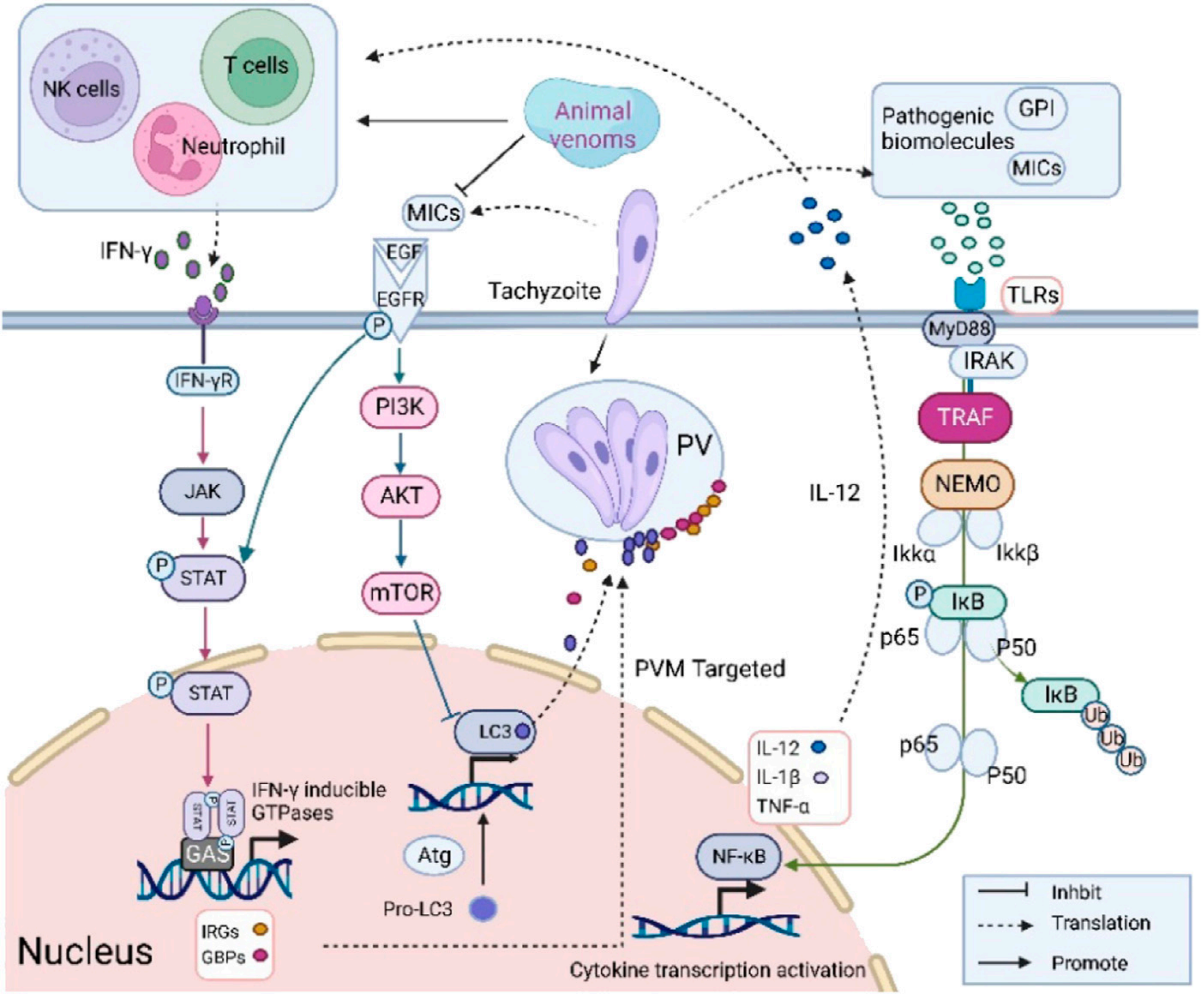
Immune response of host cells combating *T. gondii* infection. Pro-inflammatory regulators IL-12 and IFN-γ production inhibit *T. gondii* growth.

Parasite infection successively triggers innate and adaptive immune responses in the host cells (Figure 2). Firstly, innate immunity is initiated by recognition of pathogen-associated molecular patterns (PAMPs). Molecules secreted by *T. gondii* can be recognized as ligands by pattern recognition receptors (PRRs) including Toll-like receptors (TLRs), NOD-like receptors and C-type lectins (Sasai and Yamamoto, 2013). TLRs are essential for the recognition of early pathogenic organisms. Myeloid differentiation factor 88 (MyD88), an articulatory molecule located downstream of TLRs, induces transcription factors into the nucleus and regulates cytokine expression (Scanga et al., 2002). In addition, nuclear factor-κB (NF-κB) regulates the expression of inflammatory factors. It is normally found in the cytoplasm in an inactive form bound to the NF-κB inhibitor (IκB). During parasite infection, phosphorylated IκB is recognized and degraded by ubiquitin proteins. NF-κB enters the nucleus and activates transcription of inflammatory factors (Karin and Ben-Neriah, 2000). The innate immune response provides an important tool for adaptive immunity. Activation of antigen-presenting cells such as MOs and dendritic cells (DCs) induces the expression of cytokines (IL-12 and TNF-α). IL-12 stimulates the production of IFN-γ by Natural Killer (NK) cells, CD4^+^ T cells and CD8^+^ T cells (Gazzinelli et al., 1993; Sher et al., 2003).

IFN-γ produced by NK cells or other active immune cells initiates cell-autonomous immune responses which are essential for parasite defense (Brasil et al., 2017). IFN-γ can induce the proteins such as immunity-related GTPases (IRGs) and guanylate-binding proteins (GBPs) expression (Nast et al., 2020). These proteins target the PVM to remove intracellular tachyzoites. IFN-γ can also upregulate the expression of indoleamine-2,3-dioxygenase (IDO) and inducible nitric oxide synthase (iNOS). iNOS activation further stimulates NO production thereby relieving the parasite load in brain tissue (Sasai et al., 2018). Indoleamine-2,3-dioxygenase prevents parasite growth by inducing tryptophan degradation. Moreover, it has been demonstrated that the production of IFN-γ can interact with autophagy pathway, which can inhibit parasite growth through damage to PVM (Park et al., 2016).

It has been found that some animal venoms have immunomodulatory activities and inhibit the proliferation of *T. gon*dii (Castanheira et al., 2015; Borges et al., 2016; Liu et al., 2021). Here, we focus on the expression of pro-inflammatory factors following venom treatment to make reasonable hypotheses on their potential mechanism. Animal venoms suppress *T. gondii* through upregulating the expression of pro-inflammatory factors. Different concentrations of TsV promoted the expression of IL-12, IL-6 and TNF-α (de Assis et al., 2021). IL-6 is an important molecule in the inhibition of parasite growth. It can activate the JAK/STAT signaling pathway by interacting with glycoprotein 130 which can reduce the parasite burden (Silver et al., 2011). In addition, BpLec after 24 h treatment controls parasite invasion and replication by up-regulating the expression of MIF and IL-6 (Castanheira et al., 2015). MIF acts as an inflammatory stimulatory molecule that inhibits *T. gondii* proliferation and promotes macrophage phagocytosis (Ferreira et al., 2023). *T. gondii* inhibits the phagocytic capacity of MOs and drives migration to promote self-propagation (Ten Hoeve et al., 2022). Evading intracellular clearance by impairing the relevant signaling pathway of IL-12 and IFN-γ is a common regulatory strategy. Thus, venoms may act as immunostimulants to promote pro-inflammatory factor release by positively modulating Th1-type immune cells. Inhibition of inflammatory damage induced by *T. gondii* infection to maintain cellular homeostasis appears to be another viable hypothesis. Lycosin-I is a potential anti-*T. gondii* drug candidate, which downregulates IL-6 and IL-8 expression in infected cells after 48 h treatment but still stimulates pro-inflammatory factor expression in uninfected cells. Pro-inflammatory IL-8 can recruit neutrophils to inhibit *T. gondii* replication (Hayashi et al., 2003). Early inflammatory stimulus is essential for anti-*T. gondii*, but sustained stimulation can induce tissue damage such as liver injury (Lu et al., 2020). Therefore, proper inflammation control is essential for host cell protection. Li et al. showed that the mechanism of Lycosin-I relies on the phosphorylation of IκB and the blocking of NF-κB nuclear translocation (Li et al., 2018). High expression of pro-inflammatory factors inhibits parasite growth but promotes the development of apoptosis and autophagy of infected cells. Therefore, we speculate that *T. gondii* needs a suitable immune environment for intracellular parasitism. Animal venoms can upset the balance through two modes of inflammatory regulation: upregulating the expression of pro-inflammatory factors to inhibit parasite growth, and promoting the expression of anti-inflammatory factors to balance the pathological damage caused by infection.

### 4.3 Regulation of ion channel transport

**FIGURE 3:**
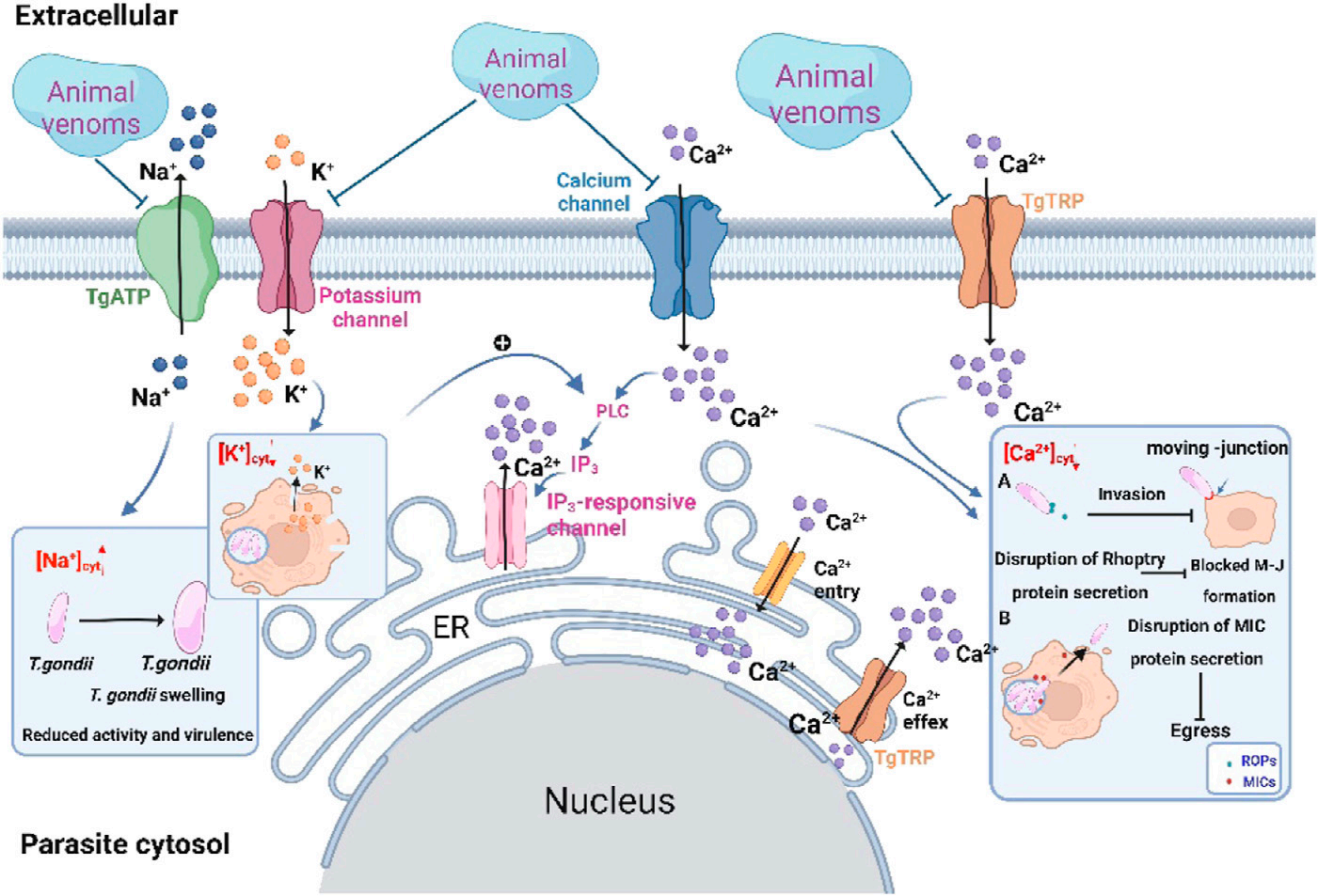
Potential model of the role of animal venoms in regulating ion homeostasis in *Toxoplasma gondii*. Venoms as channel blockers target different ion channels thereby disrupting ion homeostasis in *T. gondii*. High concentrations of Na^+^, low concentrations of K^+^ and Ca^2+^ in cytosol of *T. gondii* induce swelling of tachyzoites and inhibit invasion and egress capacity.

It is generally accepted that the key step for invasion and egress involved ion signaling (Hortua Triana et al., 2018). Under normal condition, parasites maintain differential ion concentrations of high potassium ion (K^+^) and low Na^+^ (Figure 3). Similar to mammals, the Na^+^ pump is present in the plasma membrane of *T. gondii*. Disorder in the protein encoding this ion pump inhibits the regulation of Na^+^ concentration and triggers a growth defect in parasites, which leads to parasite swelling and is reflected in reduced extracellular viability together with virulence (Lehane et al., 2019). After intracellular replication is complete, tachyzoites need to be expelled from the infected cell and invade new host cells. At this time, phospholipase C (PLC) in *T. gondii* is activated in a low-potassium environment. To balance the K^+^ deficiency leads to a further increase in intracellular calcium ion (Ca^2+^) concentration, which positively regulates parasite egress (Vella et al., 2021). The experimental results show that some animal venoms trigger morphological changes such as tachyzoites swelling and rupture of the plasma membrane. It is not known whether this swelling is caused by an increase in intracellular Na^+^ concentration and whether the membrane rupture triggers the onset of K^+^ depression. Regardless, paying attention to changes in intracellular Na^+^ and K^+^ concentrations in *T. gondii* following venom exposure may be a potential direction for mechanistic studies.

Compared to Na^+^ and K^+^, Ca^2+^ signaling has been extensively studied in *T. gondii*. The occurrence of the Ca^2+^ signaling cascade is essential for lytic cycle maintenance (Hortua Triana et al., 2018). Dysregulation of the steady-state concentration of Ca^2+^ triggers blocked secretion of the corresponding proteins in *T. gondii.* For example, TgFER2, a calcium sensor from the ferlin family, causes blocked secretion of ROPs when defective. This protein is essential for the formation of MJ (Coleman et al., 2018). Or the perforin-like protein 1 (TgPLP1) secreted by MICs, which inhibits tachyzoites egress by blocking PVM rupture in the absence of Ca^2+^ stimulation (Kafsack et al., 2009). *T. gondii* has multiple Ca^2+^ stores, such as the endoplasmic reticulum (ER), mitochondria and the plant-like vacuole, as well as Ca^2+^ channels. After replication, the influx of external Ca^2+^ and the release of stored Ca^2+^ lead to a dramatic elevation in cytosolic Ca^2+^ concentration, which is essential for successful egress (Hortua Triana et al., 2018). As a promising pharmacological blocker, this leads to the dysregulation of cytoplasmic Ca^2+^ homeostasis by inhibiting plasma membrane ion channels and Ca^2+^ efflux from calcium stores. Currently, *T. gondii* calcium-dependent protein kinase (TgCDPK) is a potential therapeutic target for *T. gondii*. As a member of the serine/threonine-protein kinase family, TgCDPK promotes MICs secretion by regulating calcium-dependent pathways. Imidazo[1,2-b] pyridazines inhibit *T. gondii* invasion by targeting TgCDPK1. It exhibited *in vitro* inhibition of the acute infection and reduced intracellular parasite burden in the micromolar range (Moine et al., 2018). In animal venoms, s-cal14.1a-mediated Ca^2+^ signaling in *T. gondii* may play essential roles in regulating parasite invasion as well as proliferation (De León-Nava et al., 2016). Researchers believe that s-cal14.1a selectively targets ion channels to inhibit tachyzoites’ cytosolic Ca^2+^ supporting. It has been shown that conotoxins interacted with voltage-gated ion channels to exert their channel-blocking effects (e.g., ω-conotoxins target Ca^2+^ channels to inhibit synaptic transmission and μ-conotoxins aim at Na^+^ channels) (Terlau and Olivera, 2004). Both s-cal14.1a and ω-conotoxins are rich in basic amino acid residues that play a key role in the inhibition of Ca^2+^ channels. Conotoxins have been extensively studied in the field of neurobiology, but further support that conotoxins function in the regulation of ion homeostasis in *T. gondii* has not yet been explored. Therefore, it is worthwhile to aim for changes in ion steady-state in tachyzoites following exposure to animal venoms.

### 4.4 Regulation of mitochondrial function

TEM results showed that tachyzoites treated with some animal-derived compounds exhibited altered in organelle arrangement and morphology (Tang et al., 2019; Liu et al., 2021). The severe disruption of organelles was detrimental to the proliferation, survival of tachyzoites as well as the feeding of nutrients. *T. gondii* has a highly dynamic double-membrane organelle, the mitochondria. This organelle controls a variety of cellular physiological activities in parasites, including energy production and metabolism, hemoglobin biosynthesis and fatty acid metabolism. These activities are closely linked to the tricarboxylic acid cycle, pyrimidine synthetic pathways and the electron transport chain (ETC) (Hortua Triana et al., 2016). In addition, significant morphological changes are induced to accommodate stimuli brought about by environmental changes. From invasion to proliferation in PV, the mitochondrial morphology of tachyzoites undergoes a transition from collapsed to lasso-like (Ovciarikova et al., 2017). TEM shows that Lycosin-I and XYP1 exacerbated the membrane permeability of tachyzoites. These two venom peptides may enter the cytoplasm through the internalization pathway, disrupting normal mitochondrial morphology (Tang et al., 2019; Liu et al., 2021). Notably, compounds from other sources confirm that mitochondrial damage plays an important role in combating *T. gondii*. For example, the natural product myrislignan induced altered mitochondrial morphology in *T. gondii* and significantly reduced adenosine triphosphate (ATP) and mitochondrial membrane potential (ΔΨm) levels in tachyzoites (Zhang et al., 2019). Monensin disrupted tachyzoite mitochondrial function and initiated oxidative stress, thereby inducing cell cycle arrest and autophagy (Charvat and Arrizabalaga, 2016). The *T. gondii* mitochondria possess more essential proteins than other organelles. These proteins are encoded by the nucleus, translated across the membranes and reached their final destination with the assistance of the translocase of the inner membrane (Usey and Huet, 2022). As most of these proteins are specific to the parasites, they are the preferred drug targets.

At present, the damage to *T. gondii* mitochondria by animal venoms is only morphologically evident, and mechanistic studies still need to be explored further. Encouragingly, there are growing evidences that the mitochondria of *T. gondii* is promising drug target. Atovaquone has an antiparasitic effect as demonstrated by Ellis in 1994 (Ellis, 1994). Atovaquone targets the cytochrome bc1 complex in the mitochondrial ETC of *T. gondii*, which was originally used for the prevention and treatment of malaria and subsequently developed into a clinical drug for toxoplasmosis. This target is associated with the oxidation of Coenzyme Q (CoQ) and hinders cell respiration by binding to the quinone reduction (Qi) or hydroquinone oxidation (Qo) sites, leading to the collapse of the mitochondrial membrane potential (Alday and Doggett, 2017). ETC is an important component of energy production in Apicomplexa parasites. Similar to humans, the mitochondria of *T. gondii* consist of multiple complexes that reduce electrons transported by substrates for eventual conversion to ATP. Notably, the type II NADH dehydrogenase (NDH2) complex is not seen in mammalian mitochondria. Saleh et al. found that 1-Hydroxyquinolone derivatives (HDQs) primarily target NDH2 for anti-*T. gondii* activity (Saleh et al., 2007).

Phenotype experiments have shown that the mitochondrial morphology of tachyzoites is altered after exposure to animal venoms (Tang et al., 2019; Liu et al., 2021). This means that the active components of the venoms are most likely to target mitochondria after crossing the membranes and entering the cytoplasm. The defective mitochondrial morphology is detrimental to the energy regulation of tachyzoites, ultimately inducing *T. gondii* death. In this sense, the active components of the venoms target and regulate the expression of key proteins that maintain the mitochondrial morphology of *T. gondii* and act as protein inhibitors to impair the energy supply. As mentioned earlier, the mitochondria of *T. gondii* are highly dynamic and can change shape in response to the intracellular and extracellular environment. After invasion, the binding of the outer mitochondrial membrane-associated protein to the inner membrane complex (IMC) of *T. gondii* is essential for the maintenance of mitochondrial morphology (Oliveira Souza et al., 2022). It has been shown that tachyzoites were unable to form a lasso-shape after invasion when the Lasso maintenance factor 1 (LMF1) was deficient, and that this protein interacted with IMC10 to maintain mitochondrial positioning and formation. In addition, mis-localization of the yeast fission protein 1 (Fis1), which is located in the outer mitochondrial membrane, also leads to abnormal mitochondrial morphology. Although these proteins may not essential for parasites, their function in regulating mitochondrial morphology is significantly lost when defective (Jacobs et al., 2020). The voltage-dependent anion channel (VDAC) is located in the outer mitochondrial membrane and is a key channel for the import of nucleus-encoded mitochondrial proteins and ions into the mitochondria. In addition to mediating the import of substances across the membrane, the channel is also a crucial site of contact between the mitochondria and the ER membrane. Studies have shown that a deficiency in this channel induced abnormal mitochondrial morphology and involved morphological changes in ER (Mallo et al., 2021).

In a word, mitochondria are considered a promising target for *T. gondii.* The mechanism by which the venoms act on the mitochondria needs to continue to be explored. Whether they can provide medicinal value by inhibiting these functional proteins and the ETC complex, and inhibit mitochondrial function by acting on proteins associated with mitochondrial morphology maintenance need to be further demonstrated in future experiments.

## 5 Future perspective

The medicinal value of animal venoms was discovered thousands of years ago and much of the current research has focused on clinical conditions such as anti-inflammatory, antibacterial and analgesic. Developments in mass spectrometry have provided us with insights into these venom components, such as peptide, toxin and protein. Some of these natural peptides with potential anti-*T. gondii* activity has significant potential for drug development. In recent years, researches about peptide therapy have increased and the evidences above suggest that animal venoms may be an effective strategy to interfere with the treatment of toxoplasmosis, but how to develop it for clinical application is a challenge. Considering that animal peptide drugs have been studied in parallel, several intrinsic problems associated with animal venom peptides have been revealed. Poor *in vivo* stability and short drug half-life lead to high plasma clearance and worse bioavailability leading to poor efficacy. XYP1 showed significant anti-*T. gondii* activity *in vivo* and *in vitro*, but was not as effective as SDZ in the plaque assay. In addition, the toxicity of the venom peptides also affects the development of peptide drugs. Therefore, although peptide drugs show great promise, there is still a need to modify peptides in a suitable way to develop them into drug peptides suitable for commercial production.

To optimize the limitations of animal peptides, researchers are constantly trying to improve strategies to enhance the stability of peptides. For example, by using different modification techniques to improve peptides properties. Firstly, peptide activity is determined by amino acid sequence, disulfide bonds and the position of the active group. Improving the activity of peptides by specific amino acid modifications is a major strategy in peptide drug development. In the case of Lycosin-I modification, the affinity of this peptide to the membrane of *T. gondii* is enhanced by the addition of arginine. Arginine is positively charged and can facilitate the interaction of the peptide with the cell membrane. Compared to the parent peptide, the modified peptide has a stronger helicity, significantly improved serum stability and significantly enhanced anticancer activity (Zhang et al., 2017). In addition to amino acid modifications, mimetic peptides, amino acid cyclization, lipidation and truncation are all promising for development (Li et al., 2021). Truncating the length of the peptide while retaining the active group ensures high affinity and specificity between the peptide and the target protein on the parasite. In addition, the selection of suitable drug delivery systems can reduce serum clearance and improve efficacy. The main advantages are increased stability and bioavailability, reduced impact of degradation enzymes and specific target release. The cellular transport challenge can address by the introduction of nanoparticles, which are delivery platforms to facilitate the delivery of peptide biologics and the treatment of various inflammatory diseases. The combination of peptides with other substances can significantly improve drug attributes. For example, Nanoscale drug delivery systems (Nano-DDS) improve the permeability of peptide molecules by protecting them from degradation and delivering them to specific sites (Radaic et al., 2020).

Finally, it is worth noting that current animal venom studies have focused on tachyzoites and are focused on their effect during the lytic cycle of invasion and replication. However, most of the strains isolated from the clinic are type II strains causing latent infections, which differ significantly from the type I strains used in the experiments regarding virulence and viability (Ajzenberg et al., 2002). Cysts in the brain and muscle are currently poorly treated with drugs. Therefore, the development of animal venoms for the treatment of cyst formation provides better clinical value.

## 6 Conclusion

In the face of the shortcomings of current toxoplasmosis treatments, researchers still need to find compounds with low toxicity and good activity. Desirable drugs also require effective tissue cysts penetration, but drugs with fetal toxicity must be excluded. Venom-isolated molecule therapy is effective in the treatment of diseases such as clinical hypertension and diabetes. Therefore, it is a promising choice to consider animal venom as a potential candidate for drug development.

From the point of view of safety and efficacy, venom peptides are promising candidates as they can exert anti-*T. gondii* activity in small doses and in an efficient manner. The clinical medicinal dose of SDZ is 400μΜ (100 μg/mL). Most animal venoms significantly inhibit the invasion and proliferation of *T. gondii* at low doses (Table 1). The survival time of HWVM-treated mice was 5 days longer than that of SDZ-treated. In addition, animal venoms contain a wide range of bioactive substances such as peptides, polyamines and proteins. *T. gondii* plasma membrane integrity critical to invasion. Peptides with a α helical structure can target specifically on the membranes of bacteria and parasites, causing membrane damage. This action mode may help prevent the development of drug resistance. Furthermore, the natural animal venom has an immunomodulatory property. *T. gondii* can evade the host immune system and causes life-long infection. The animal venom is an immunostimulant to enhance the host immune response and promote parasite intracellular clearance. Finally, the lytic cycle of *T. gondii* cannot be regulated without ion homeostasis. Calcium signaling activation is involved in multiple processes as well as supports parasite growth. Animal toxins as well as peptides have good channel targeting effects which may be potential candidates for channel inhibitor.

Since ancient times, Venom-derived molecule therapy has been used for the treatment of various inflammatory diseases, cancer and other diseases. In modern times, it has become more systematic and rigorous. A variety of venom peptides of animal origin have been approved for clinical use, such as exenatide, ziconitide and captopril. Finally, advancements in mass spectrometry and molecular biology techniques are making the future of animal venom research even more promising. Thus, we believe that animal venom is a wonderful source of anti-*T. gondii* drug candidates.

## Figures and Tables

**Table:** 

***T. gondii***	*Toxoplasma gondii*
**SDZ**	Sulfadiazine
**PYR**	Pyrimethamine
**PLA2**	Phospholipase A2
**SEM**	Scanning Electron Microscope
**TEM**	Transmission Electron Microscope
**PVM**	Parasite vacuole membrane
**IC**_**50**_	Half maximal Inhibitory Concentration
**IL-6**	Interleukin-6
**MIF**	Macrophage migration inhibitory factor
**TGF-β1**	Transforming growth factor-β1
**TNF**	Tumour Necrosis Factor
**IFN-γ**	Interferon-γ
**NO**	Nitric Oxide
**ROP**	Rhoptry protein
**PV**	Parasitic vesicle
**IMC**	Inner membrane complex
**PAMP**	Pathogen-associated molecular pattern
**TLR**	Toll-Like Receptor
**DC**	Dendritic cell
**MyD88**	Myeloid Differentiation Factor 88
**NF-κB**	Nuclear factor-κB
**STAT**	Signal transducer and activator of transcription
**JAKs**	Janus-activated kinase
**GAS**	Gamma-activated site
**GBP**	Guanylate-binding protein
**ROS**	Reactive oxygen species
**iNOS**	Inducible nitric oxide synthase
**ER**	Endoplasmic reticulum
**Na** ^**+**^	Sodium ion
**K** ^**+**^	Potassium ion
**Ca** ^**2+**^	Calcium ion
**PLC**	Phospholipase C
**MJ**	Moving junction
**CDPK**	Calcium-dependent protein kinase
**ETC**	Electron transport chain
